# Dr. F. J. Waldo's Experiences in 1914

**Published:** 1915-05-15

**Authors:** 


### A CORONER'S REPORT.

### Dr. F. J. Waldo's Experiences in 1914.

The Coroner's inquest is not an institution of which all men speak well. Neither on its legal side nor in its medical relations has it escaped criticism. And somewhere, in an official pigeon-hole, rests the report of a relatively recent Departmental Committee which urges its amendment. Nevertheless, so decided a fund of virtue does it possess that it has continued to exist and to enjoy a large share of public confidence during some eight hundred years. It may fail to satisfy alike the lawyers and the doctors, but the English people generally will not lightly let it go. It is a feature in the life of the nation, and is widely regarded as a useful agency for the detection of anything in the shape of foul play.

Apart from its legal value, whatever this may be, a record of the work of a Coroner has a certain social and economic interest. It reveals phases of life which to many people are almost unknown. For the most part its searchlight is turned upon uninviting incidents and exhibits tragedies in the less happy area of the nation's life. This is more especially true where the jurisdiction involved is the crowded district of one or other of our great cities. Yet it is here, we venture to think, that the value of the Coroner's inquest is most fully seen, just as it is here that the conduct of these inquiries reaches its most efficient expression. An illustration of this proposition may be found in the recently issued report of Dr. F. J. Waldo, His Majesty's Coroner for the City of London and Borough of Southwark.

In the year 1914 Dr. Waldo conducted no fewer than 412 inquests, and in 148 of these the death of the person in question was ascertained to be due to natural causes, while in the remaining 264 the cause of the fatal event was either unnatural or could not confidently be determined for lack of evidence. In this latter group 199 deaths resulted from accident, thirty-six from criminal acts, eight from excessive drinking, and five from want and exposure; in thirteen cases an "open verdict" was returned. The fatalities due to vehicles numbered forty-six, and of these thirty-six were caused by mechanically propelled vehicles and ten by vehicles drawn by horses. In the City every ninth, and in Southwark every fourth inquest, apparently, is held upon the body of a child under the age of seven years. Of the criminal deaths one was that of a street newsvendor shot by a man who had shortly before been discharged from a lunatic asylum, and who used his liberty to purchase the pistol with which he promptly took the life of an inoffensive stranger. Another case was that of an "unfortunate" woman, who was found dead, with terrible injuries on her head and body, in a wretched room said to be her home. The district in which this murder occurred is described as a breeding ground of crime, drink, immorality, filth, and disease, and from it frequent violent and unnatural deaths, both of adults and children, come for investigation to the Coroner's Court. Such is a rapid glance at a picture of certain aspects of the national life as it exists in the centre of the richest city in the world in the early years of the twentieth century. It gives some cause for thought, and one may wonder what the student of social history in the year 2500 will say about it.

One of the privileges of the Coroner's jury, in delivering its verdict, is to add a "rider," in which an oblique censure may be passed on someone more or less closely related to the fatal story under investigation, or advice may be tendered with the hope of preventing similar disasters in the future. These "riders" have no legal efficacy and they make no pretence to express expert opinion. But there is often a robust common sense about them, and they frequently say what many people think. In the present report we find them dealing with such questions as the provision of street refuges, the lighting of the streets, and the regulation of traffic as these arrangements bear upon the occurrence of street accidents; also the conditions which govern the sale and storage of poisons and the dispensing of these substances in public institutions; and the proper equipment of workshops, with facilities for the escape of the workers in the case of a fire, and so on. These riders may often claim to be a fair index of public opinion, and they must help to keep up a standard of efficiency where otherwise carelessness and selfishness might prevail.

Dr. Waldo has himself in his report many suggested improvements to propose on the basis of experience gathered in his court. He finds, for example, a deficiency in the Pistols Act, since a weapon having a barrel exceeding nine inches in length is not a "pistol" in the legal sense, and can therefore be sold to a youth under eighteen years of age. All the same, such a firearm will send a bullet through three inches of deal, and as a matter of fact was used for the shooting of two persons by a boy of sixteen years in 1914. In connection with motor traffic, Dr. Waldo would have punishment by imprisonment applied not only to a driver who was "actually drunk," but also to one who had unfitted himself for his work by being "under the influence of drink." He advocates, too, the compulsory adoption of lifeguards, such as are used on omnibuses, by all similar vehicles, but apparently it is not easy to discover the authority who could be set into operation to this end. Indeed, there is a still wider uncertainty, for in Dr. Waldo's attempts to secure an increase in the number of street refuges he addressed himself to the County Council, who duly referred him to the Borough Councils, by whom he was politely bowed back again to Spring Gardens. And yet we had fondly believed that we had succeeded in simplifying the municipal administration of the Metropolis! We hope the day is not distant when Dr. Waldo will find time to give us a summary of his special experience and a reasoned account of the medico-legal and economic lessons which it teaches.

